# Supplementary figures and images for: A curated census of pathogenic and likely pathogenic UTR variants and evaluation of deep learning models for variant effect prediction
Source: Front Mol Biosci. 2023 Sep 8;10:1257550. doi: 10.3389/fmolb.2023.1257550 (PMC10517338; doi:10.3389/fmolb.2023.1257550)

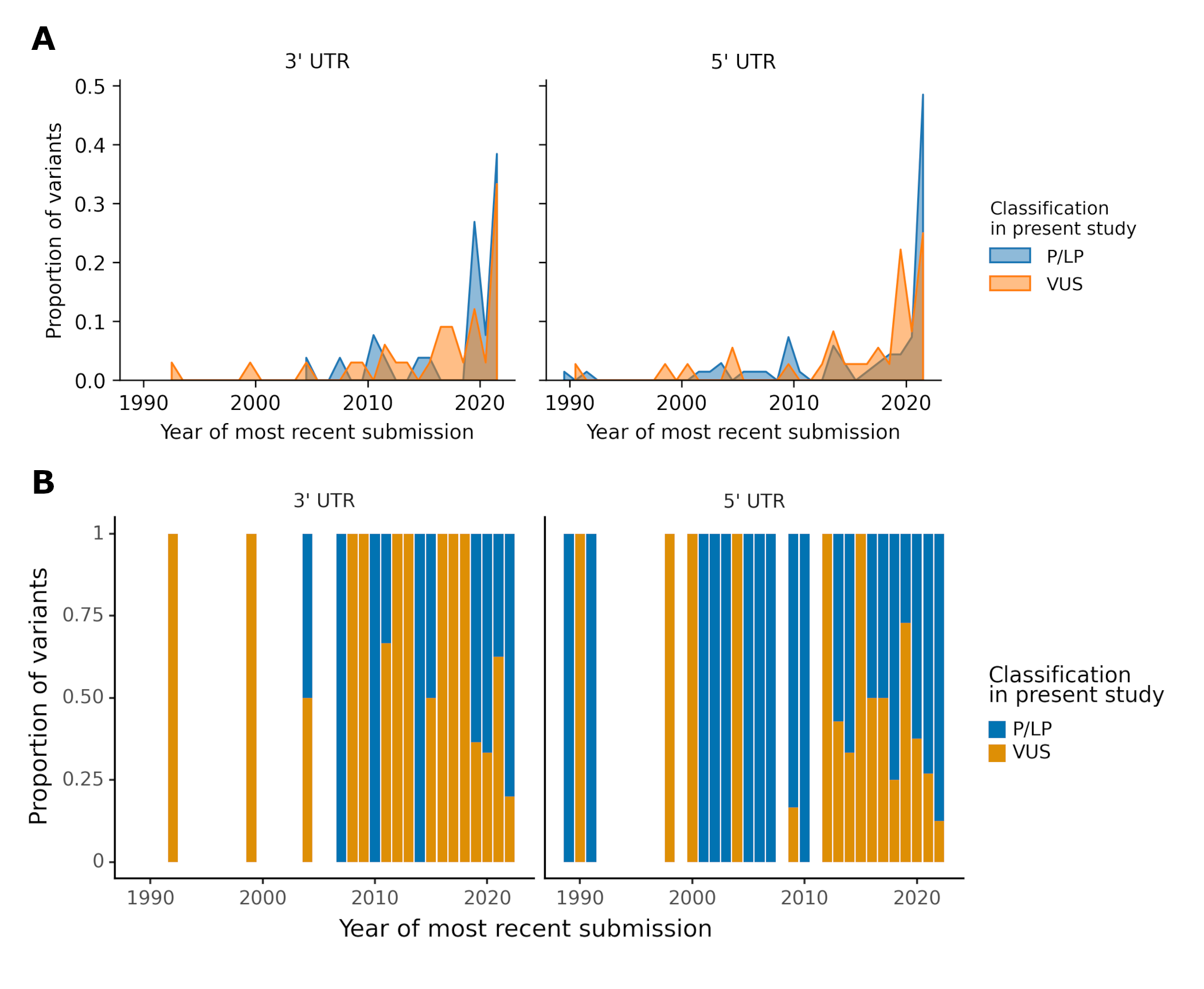

Supplement: Supplementary file 1 [file Image3.TIFF]

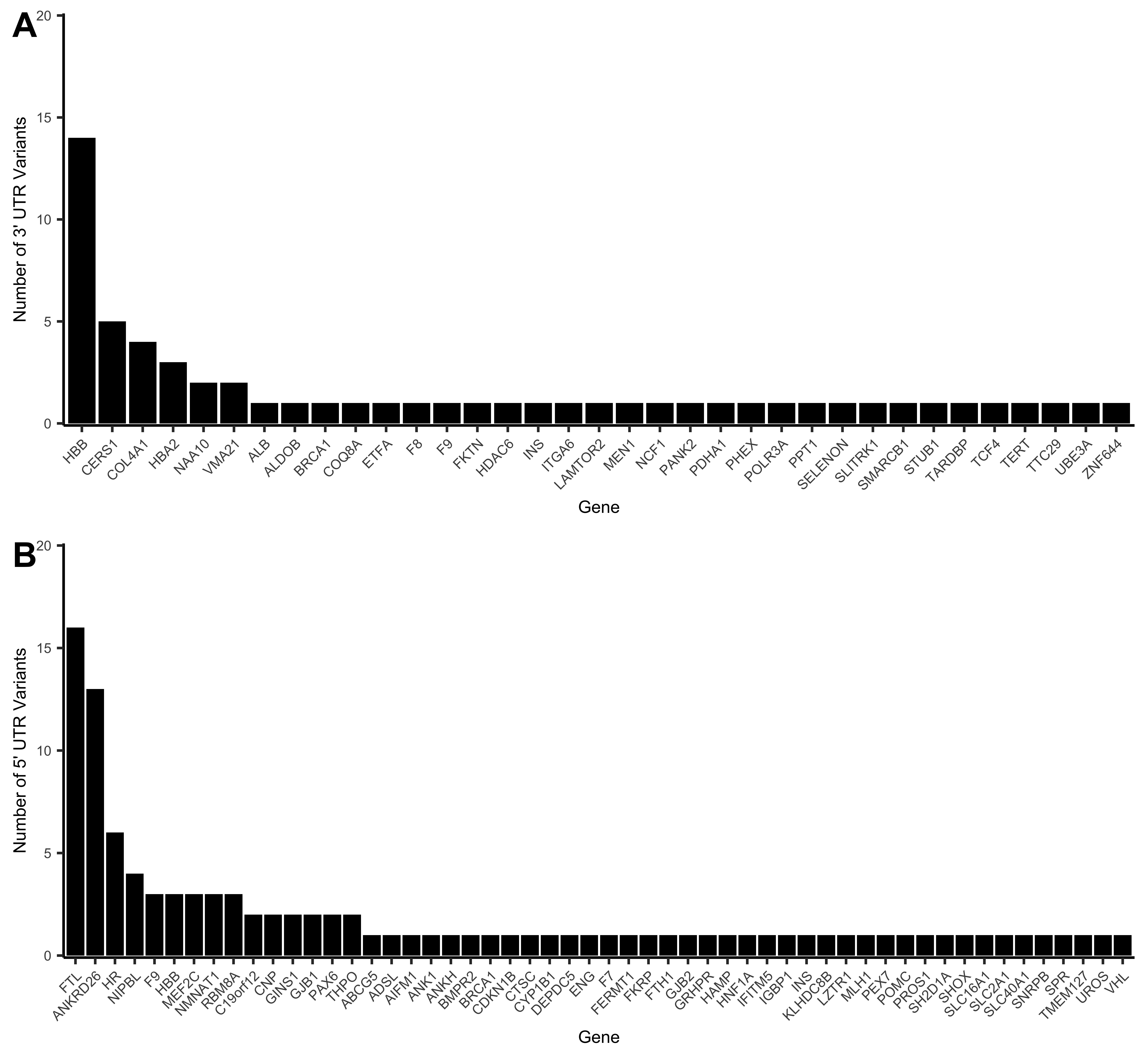

Supplement: Supplementary file 3 [file Image1.TIFF]

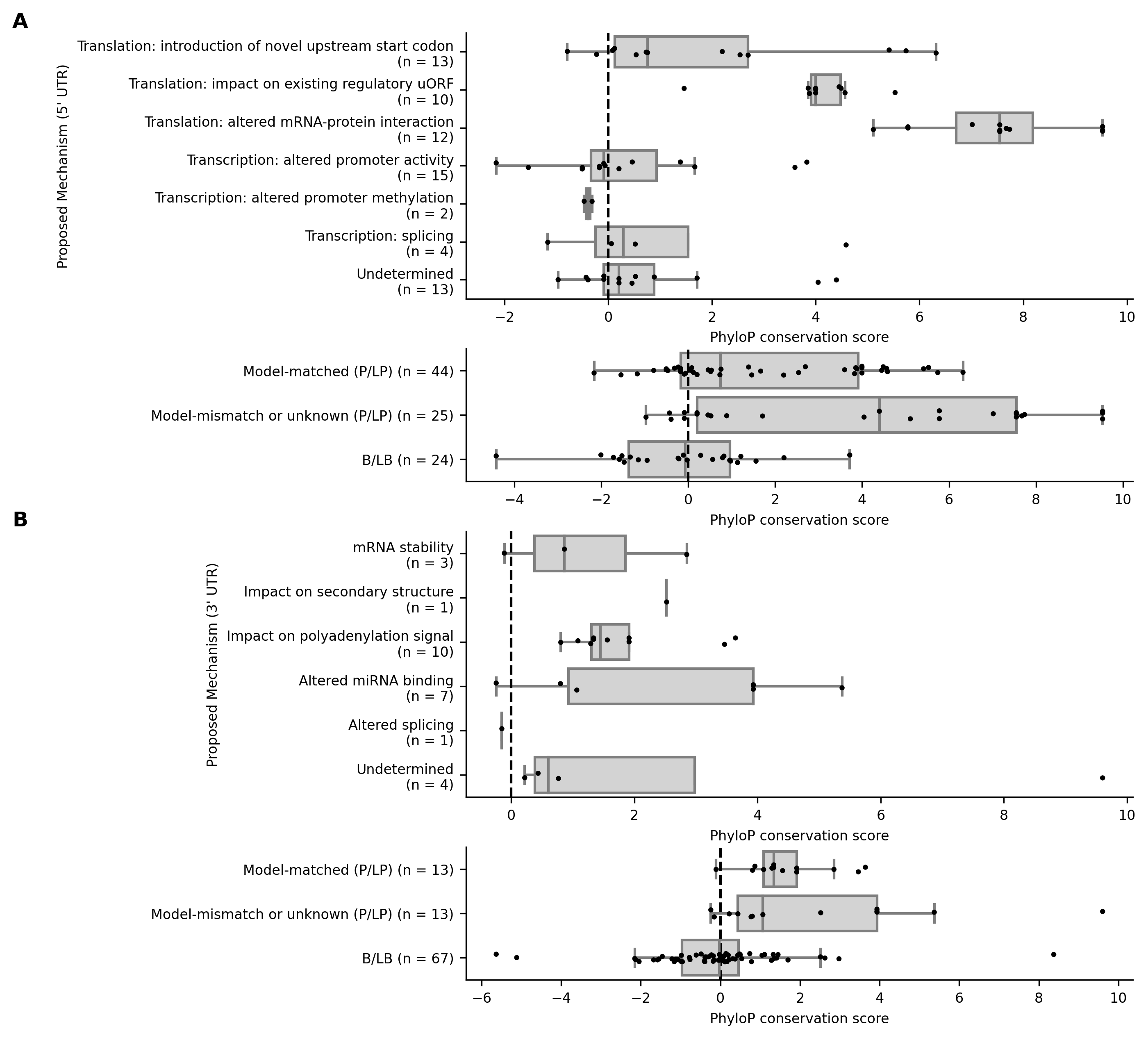

Supplement: Supplementary file 4 [file Image9.TIFF]

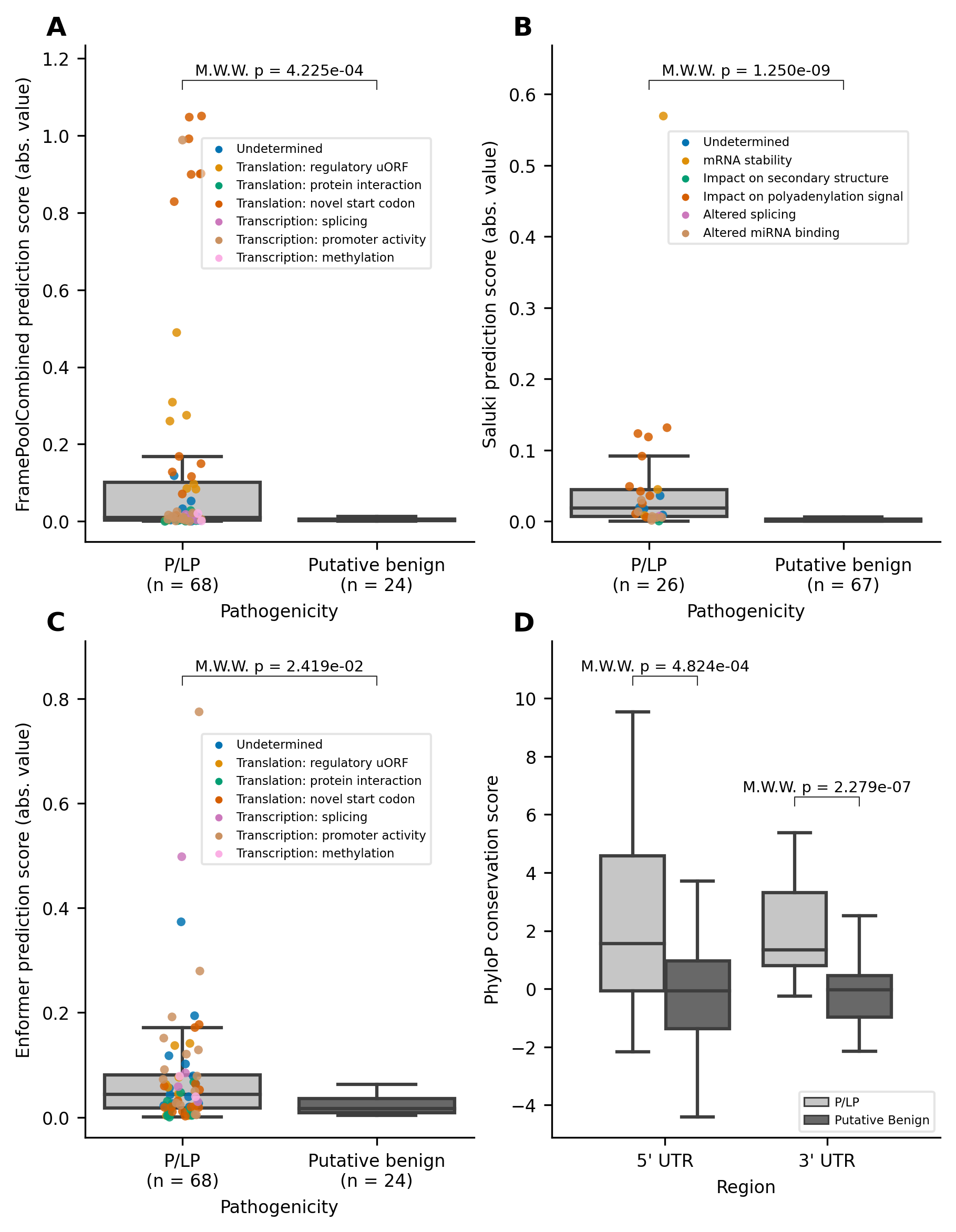

Supplement: Supplementary file 6 [file Image5.TIFF]

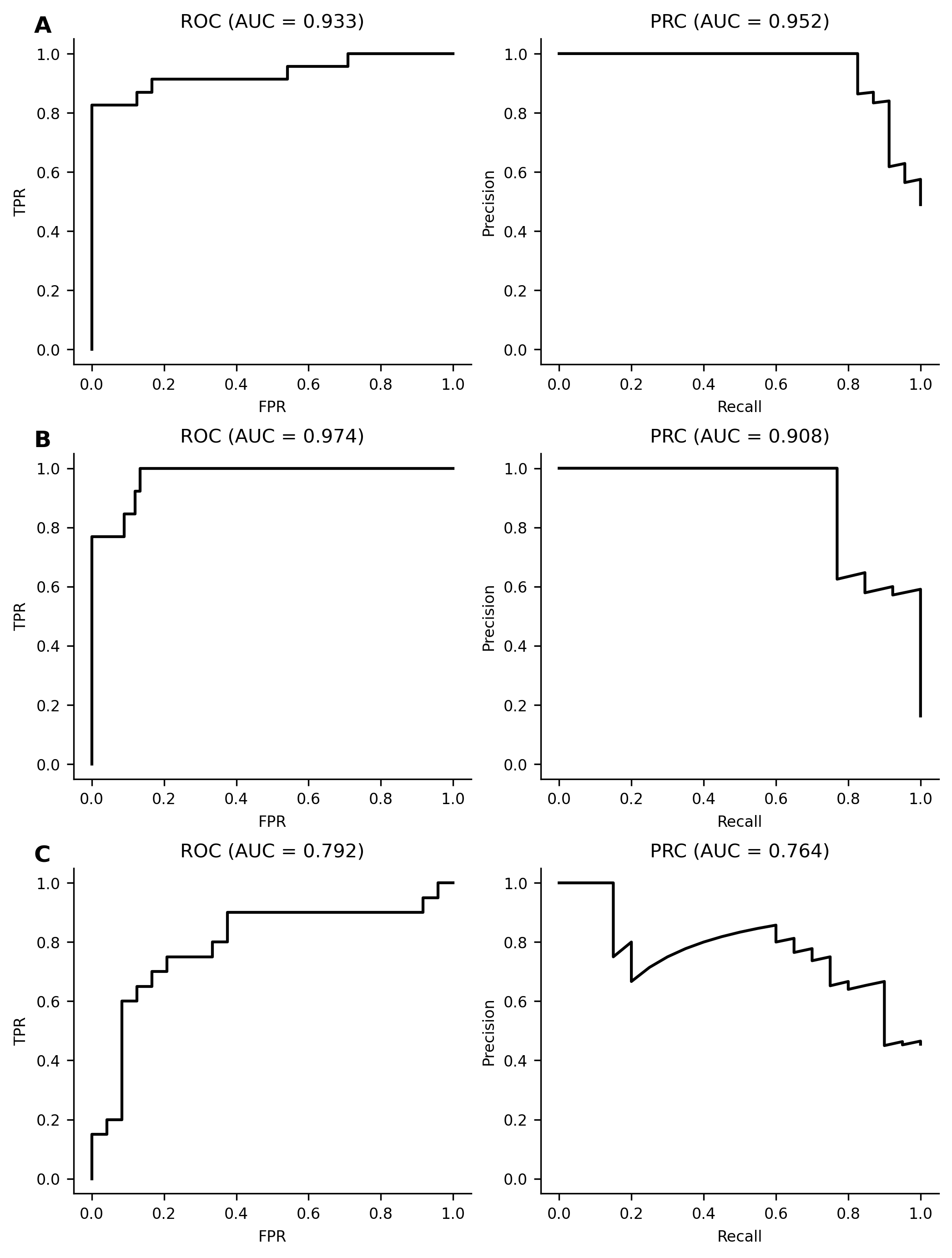

Supplement: Supplementary file 7 [file Image8.TIFF]

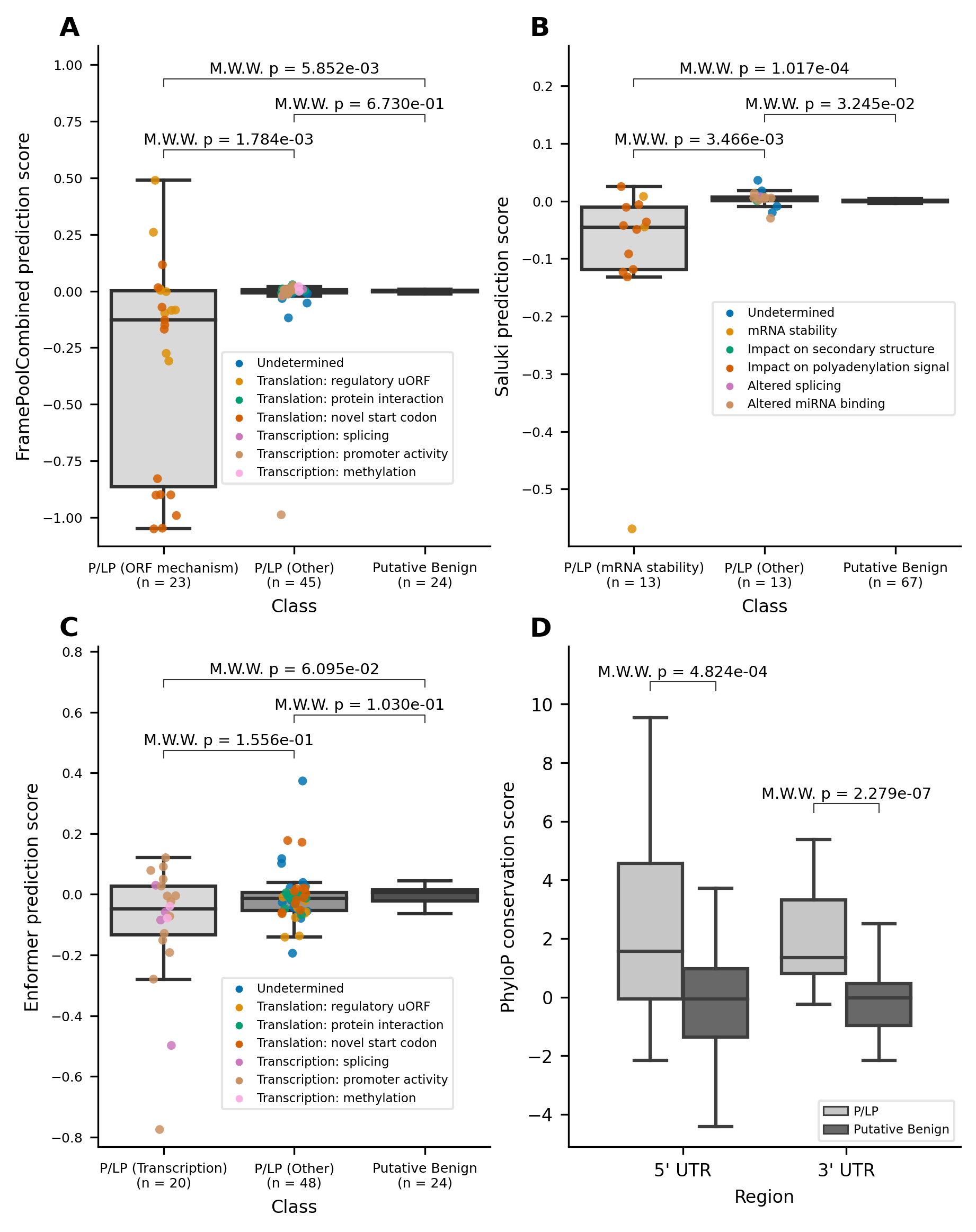

Supplement: Supplementary file 11 [file Image6.TIFF]

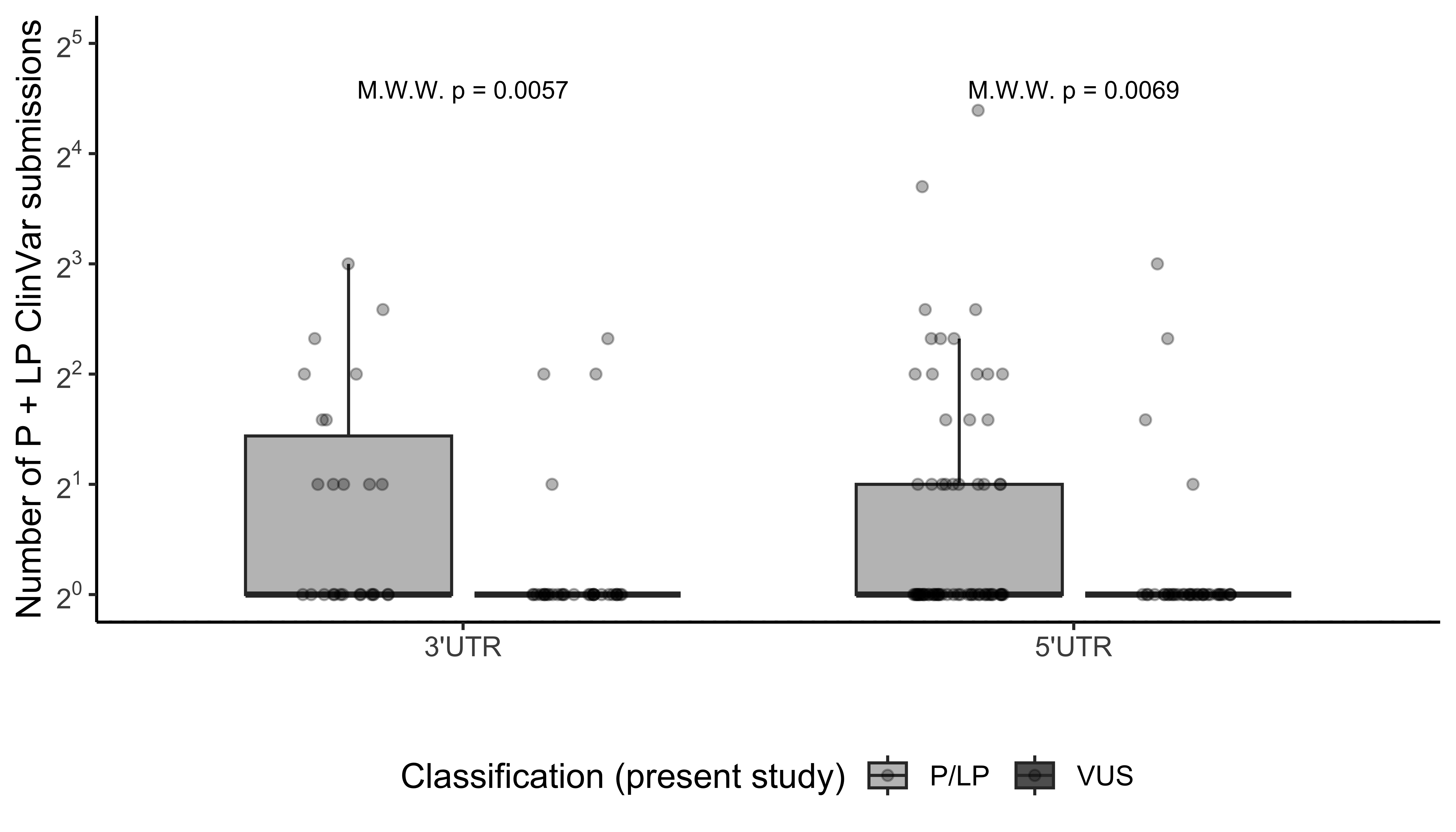

Supplement: Supplementary file 13 [file Image2.TIFF]

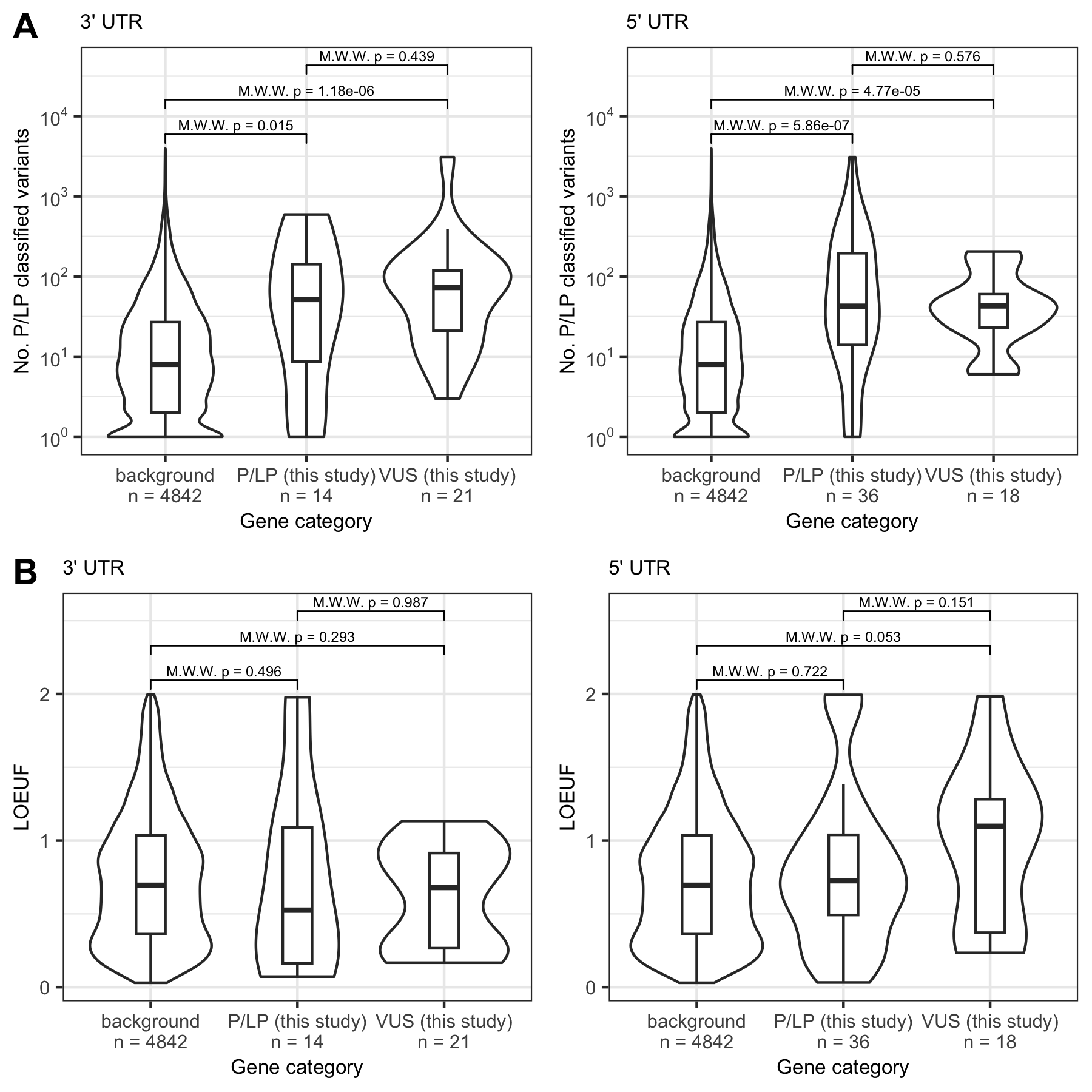

Supplement: Supplementary file 14 [file Image4.TIFF]

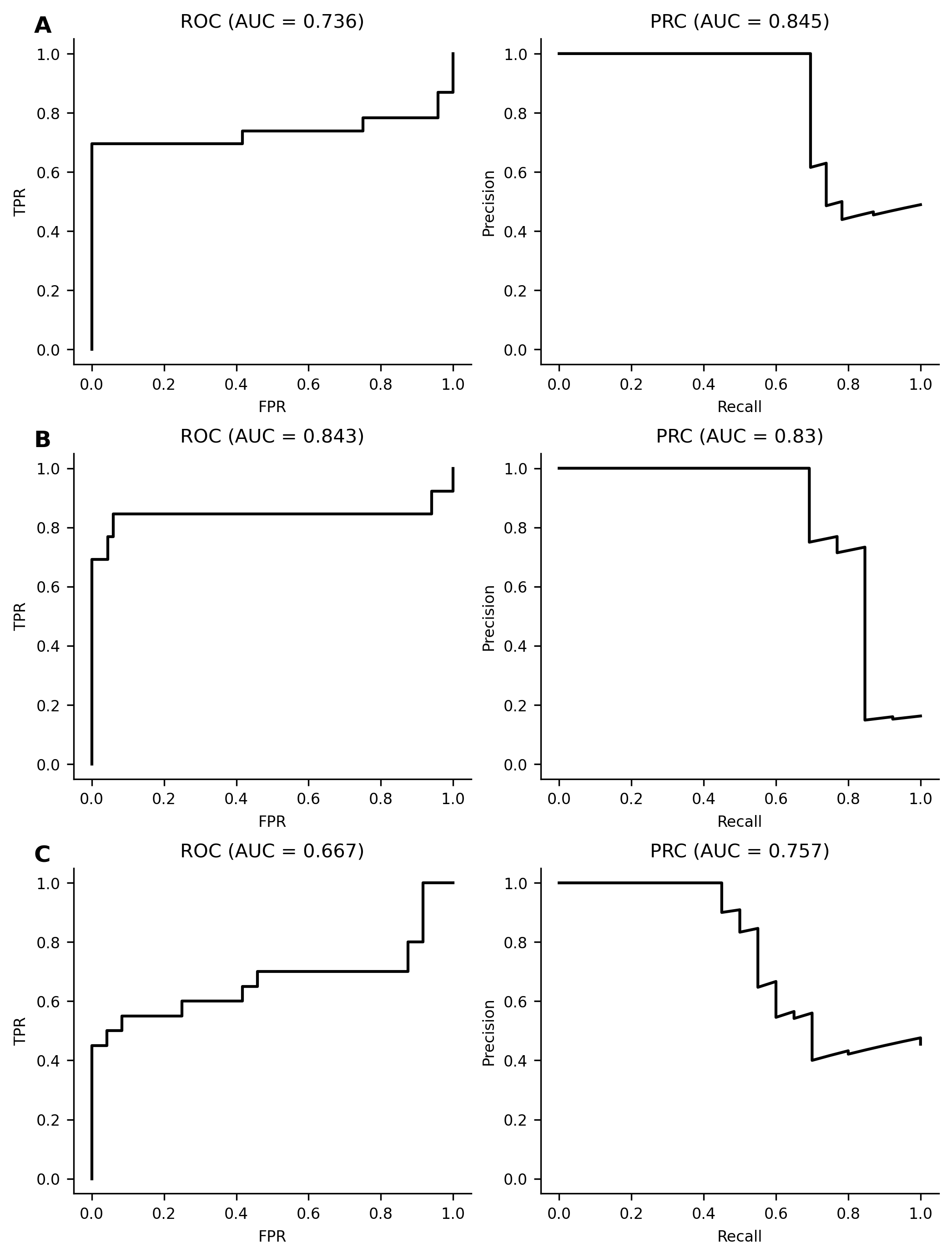

Supplement: Supplementary file 15 [file Image7.TIFF]
